# Brain death and changes in solid organ characteristics due to acute lack of female sex hormones

**DOI:** 10.1186/cc14692

**Published:** 2015-09-28

**Authors:** Roberto Armstrong Junior, Ana Cristina B Faloppa, Guilherme K Kudo, Luiz Felipe P Moreira, Paulina Sannomiya, Raif R Simão, Sueli G Ferreira

**Affiliations:** 1 Heart Institute (INCOR), University of São Paulo Medical School, São Paulo, SP, Brazil

## Introduction

Previous studies have suggested that female sex hormones have a protective action, because they contribute to reduce the inflammatory degree in females after trauma.

## Objective

This study aimed to investigate sex differences in the course of the inflammatory process in rats subjected to brain death (BD).

## Methods

Wistar rats were randomized into three groups (male rats, *n *= 5; female rats, *n *= 10; and ovariectomized rats, *n *= 5) and subjected to BD by rapid inflation of a catheter Fogarty^® ^4F. The liver, kidneys, lungs and the heart were collected after 6 hours and samples (4 µm) were stained with H&E for histological analyses. Leukocyte infiltration, edema and hemorrhage were measured and data were compared using GraphPad Prism v.6.10, and *p *values lower than 0.05 were considered significant.

## Results

Female rats exhibited increased leukocyte infiltration into the lungs and the heart when compared with male rats (*p *= 0.009 in the lungs and *p *= 0.022 in the heart) and presented also a sudden decrease in estradiol levels 6 hours after BD (*p *= 0.01). The intensity of hemorrhage was greater in ovariectomized rats compared with the other groups (*p *= 0.001) in the lungs. All groups presented slight to moderate leukocyte infiltration and absence to slight hemorrhage in the liver. Leukocyte infiltration had a wide distribution in female rat kidneys, and in male and ovariectomized rat kidneys infiltration varied from absent to slight.

## Conclusion

The increased inflammation in the lungs and heart of female rats might be a result of the lack of female sex hormones. Therefore, the idea of introducing a therapeutic use of female sex hormones on female BD donors could be considered.

